# Conquering Challenges: A Case Series of Yellow Phosphorus Poisoning at a Tertiary Care Hospital in Pune, Western India

**DOI:** 10.7759/cureus.61416

**Published:** 2024-05-31

**Authors:** Nikhil I Doshi, Kishor M Khillare, Bhumika Vaishnav, Ruchitha Pailla, Suvidha Shaha

**Affiliations:** 1 General Medicine, Dr. D. Y. Patil Medical College, Hospital & Research Centre, Pune, IND; 2 Psychology, Ajeenkya DY Patil School Of Engineering, Pune, IND

**Keywords:** toxicology, poisoning, acute liver failure, rodenticides, yellow phosphorus

## Abstract

Rodenticides are easily available in the market and suicidal attempts by ingesting such poisonous products are commonly reported in rural India. We aimed to analyze predictive factors, biological markers, and treatment outcomes among patients who ingested rodenticides (yellow phosphorus) with the brand name, Rattol. Here, we present three such cases who were admitted to a tertiary care hospital. We recorded socio-demographic characteristics, probable predictive factors, and serial charting biological markers. Conventional treatment was given to these cases. All cases were young women (age range: 17-30 years) from rural areas, two were married and one was unmarried. The approximate quantity of ingestion was 20, 10, and 5 grams, respectively. The time lag between the ingestion and sought first health care was 6 hours, 18 hours, and 1 hour, respectively. Major symptoms were vomiting, abdominal pain, and headache. Biological markers, including total bilirubin, alanine aminotransferase, aspartate aminotransferase, creatinine, prothrombin time, international normalized ratio, and model for end-stage liver disease (MELD) score were statistically significant. Two women had toxic hepatitis and acute liver failure and one did not have any organ damage. All of them were recovered within 17 days of mean hospital stay. A lethal dosage of rodenticides and delayed presentation to the hospital can prompt acute liver failure and severe ailment. Creating awareness, promoting mental health and suicide prevention, and framing proper guidelines for treatment will reduce morbidity and mortality.

## Introduction

Rodenticides are broadly showcased in India with different formulations that are accessible, like Rattol (yellow phosphorus), as paste and powder. It is cheap, effectively accessible over the counter, and online web-based business destinations in India. There are frequently reported cases of suicides due to rodenticide ingestion, especially in rural areas of India [1]. Accidental consumption of rodenticide paste is not uncommon among children. For a long time, rodenticides have been a significant reason for morbidness and mortality in patients who present to a trauma center with purposeful self-hurt [2]. In India, self-destructive or unintentional harming with rodenticide containing yellow phosphorous is a more successive reason for drug-prompted acute liver failure than paracetamol [3]. Various systems can be affected like the hepatic, renal, cardiac, and central nervous system, so daily clinical evaluation should be done [4,5]. A study conducted at a tertiary care hospital in South India showed that yellow phosphorus was a common rodenticide used in suicide attempts with >76% mortality despite maximal supportive therapy [6]. Although more research has been done to lay out medications and drugs for the treatment, no specific antidote has been recognized against that. Therefore, the main treatment remains supportive care.

## Case presentation

Here, we present three case studies of patients who ingested rodenticide (yellow phosphorus) and were admitted to a tertiary care hospital. Case 1 was a 28-year-old unmarried, graduate, and unemployed female. Case 2 was a 30-year-old, married, literate female who was a homemaker. Case 3 was a 17-year-old, unmarried female student.

Table 1 lists the history of patients with their chief complaints, the first healthcare provider the patients visited, the mode of ingestion of rat killer poison, the quantity of the poison they ingested, and the first healthcare-seeking time.

**Table 1:** Clinical presentation to tertiary care hospital on the day of admission

Related profile of ingestion of rat killer poison	Case 1	Case 2	Case 3
1st healthcare visited	Local clinic, Pune	Aurangabad GMC	D.Y. Patil Hospital, Pune
Mode of ingestion of rat killer poison	Paste	Paste	Mixed with food
Quantity ingested (in grams)	20>=	10>=	5<=
1^st^ healthcare seeking time (in hours)	6	18	1
Most common major symptoms*	Vomiting, abdominal pain, headache	Vomiting, abdominal pain, headache	Vomiting, headache

Patients were screened for clinical findings. Demographic and biological markers were collected and analyzed.

Table 2 shows hematological investigations on the day of admission: total protein, total bilirubin, direct and indirect bilirubin, alanine aminotransferase (ALT), aspartate aminotransferase (AST), alkaline phosphate (ALP), platelet count, albumin, urea, creatinine, prothrombin time (PT), and international normalized ratio (INR) were done with serial charting of biological markers.

**Table 2:** Biological markers on the day of admission Hb: hemoglobin, TLC: total leukocyte count, AST: aspartate transferase, ALT: alanine aminotransferase, PT: prothrombin time, INR: international normalized ratio, MELD: model of end-stage liver disease

Value biomarkers (on admission)	Reference range/unit	Case 1	Case 2	Case 3
Hb	13.2 – 16.6 gm/dL	10.5	11.4	13.5
TLC	4000 – 10000 /µL	7000	6000	5400
Platelet	150000 – 410000/µL	370000	128000	223000
Total bilirubin	0.22 –1.20 mg/dL	13.3	8.86	0.44
AST (U/L)	8 – 48 U/Lt	1066	484	10
ALT (U/L)	7 – 55 U/Lt	337	352	18
Total protein	6.4 – 8.3 g/dL	6.9	5.6	7.5
Albumin	3.5 – 5.2 gm/dL	4	3.5	4.2
Urea	17 – 49 mg/dL	38	25	18
Creatinine	0.6 – 1.35 mg/dL	0.58	0.5	0.84
PT	11 – 13.5 seconds	38	35.8	12.3
INR	0.8 – 1.1	3.5	3.1	1.07
MELD	-	30	25	7

Serial charting of biological marker assessment

Complete blood count and serum electrolytes were within normal limits for all three cases.

**Figure 1:**
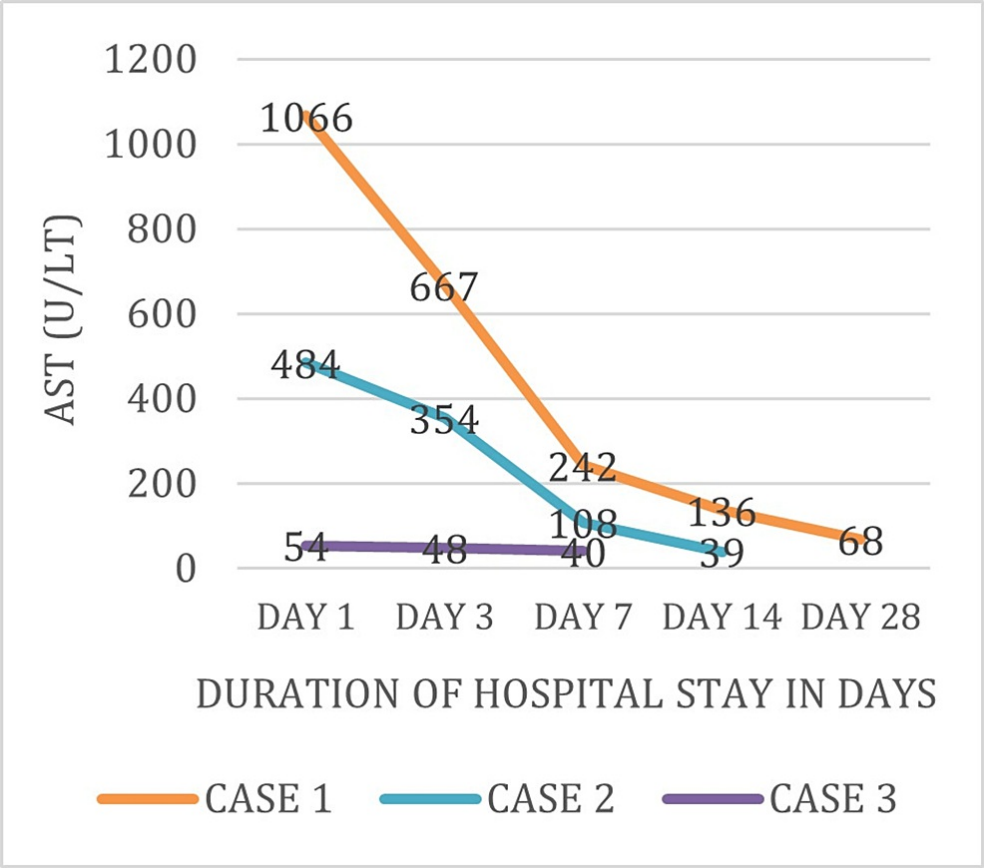
Trend of AST levels during the course of hospital stay AST: aspartate aminotransferase

The following figures are a comparative graph of biological markers (AST, ALT, PT, INR, and model for end-stage liver disease (MELD) score) for the duration of hospital stay. It can be observed that deranged levels of biological markers were near the normal range at the time of discharge. Figure 1 shows the AST levels of three patients (Cases 1-3) were 1066, 484, and 54 units/liter respectively on day 1 of admission. These levels were further decreased on subsequent days of admission and were near normal at the time of discharge.

**Figure 2:**
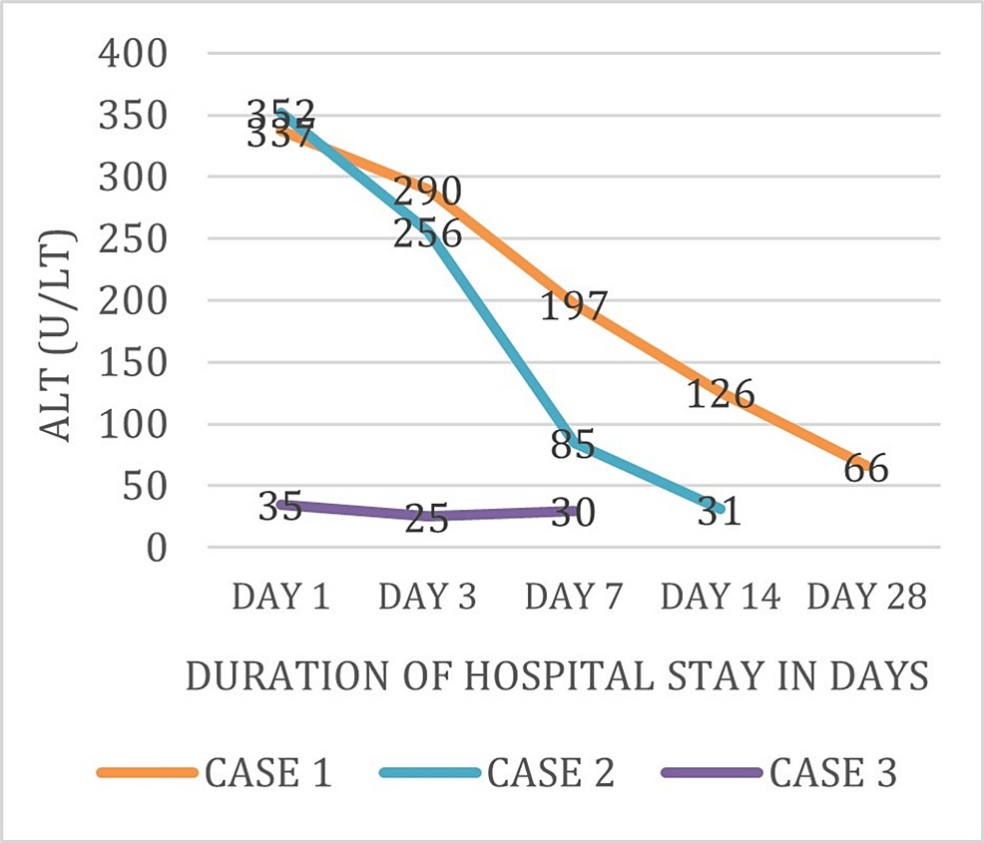
Trend of ALT levels during the course of hospital stay ALT: alanine aminotransferase

Figure 2 shows the ALT levels of three patients (Cases 1-3), which were 337, 352, and 35 units/liter respectively on day 1 of admission. These levels were further decreased on subsequent days of admission and were near-normal at the time of discharge. 

**Figure 3:**
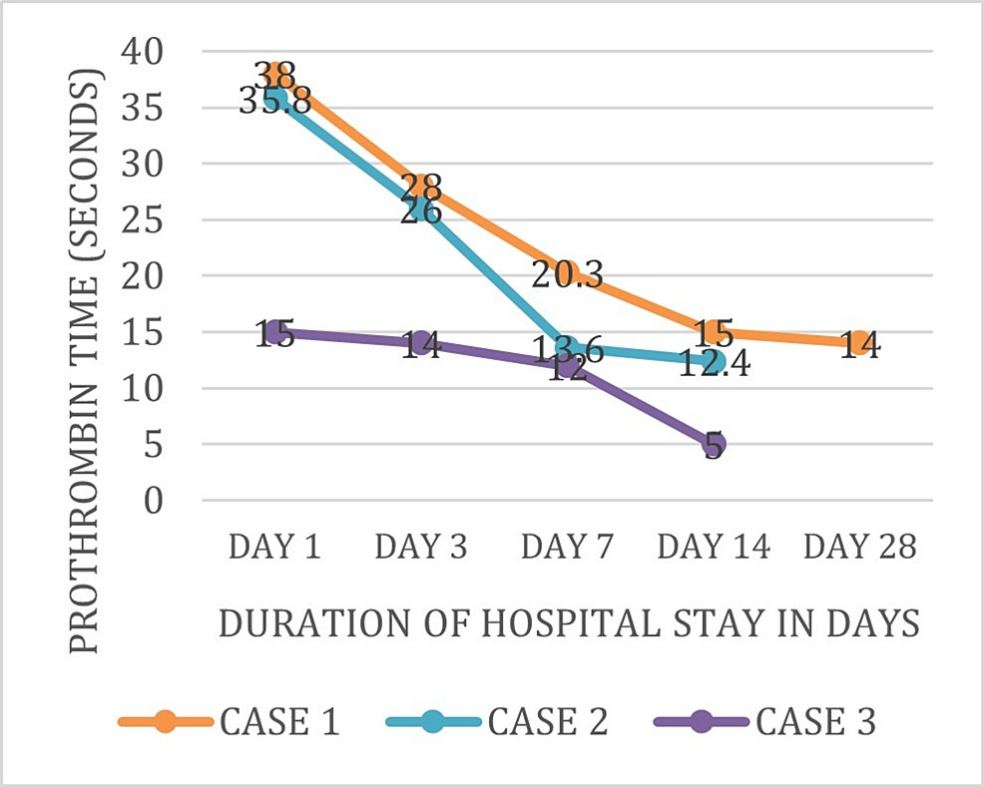
Trend of prothrombin time during the course of hospital stay

Figure 3 shows the PT levels of three patients (Cases 1-3) were 38, 35.8, and 15 seconds, respectively, on day 1 of admission. These levels were further decreased on subsequent days of admission and were near normal at the time of discharge.

**Figure 4:**
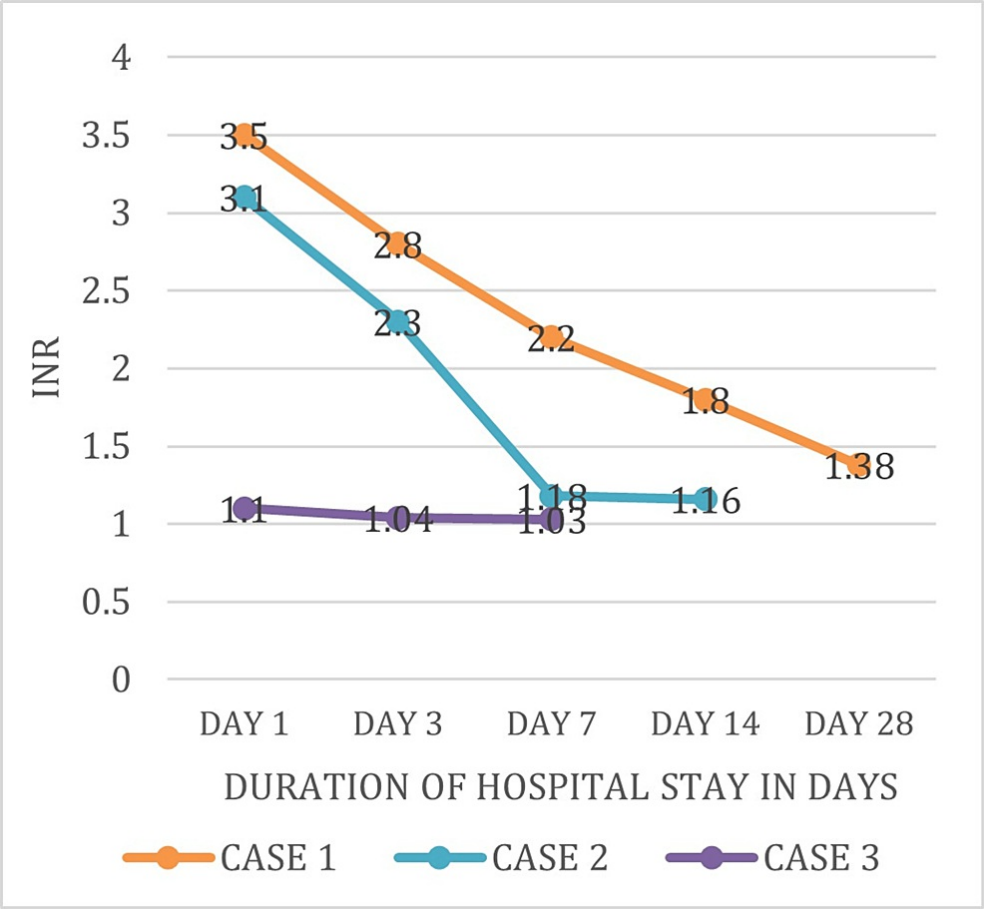
Trend of INR values during the course of hospital stay

Figure 4 shows the INR levels of three patients (Cases 1-3) were 3.5, 3.1, and 1.1 respectively on day 1 of admission. These levels were further decreased on subsequent days of admission and were near-normal at the time of discharge.

**Figure 5:**
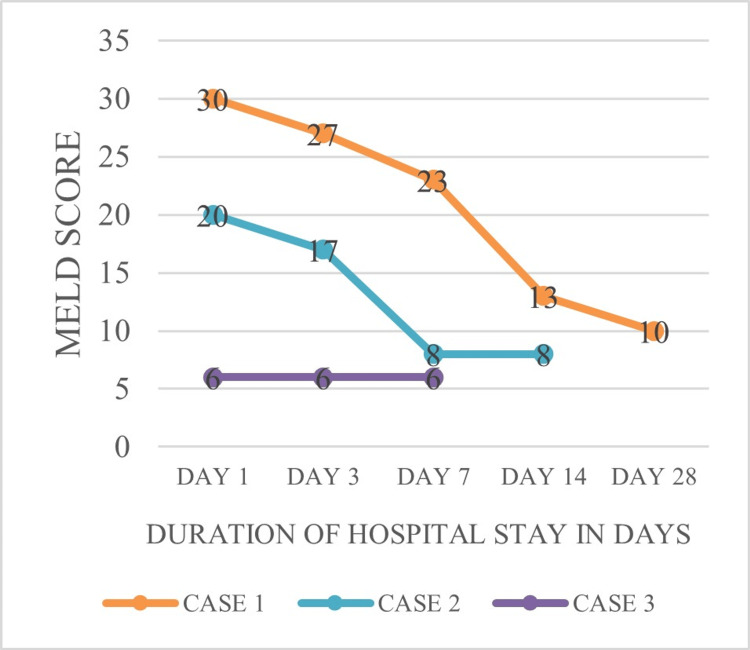
Trend of MELD scores during the course of hospital stay MELD: model of end-stage liver disease

Figure 5 shows the MELD scores of three patients (Cases 1-3) were 30, 20, and 6, respectively, on day 1 of admission. These levels were further decreased on subsequent days of admission and were near normal at the time of discharge.

Results

In our cases, the patients ingested rodenticide (yellow phosphorus), approximately 20, 10, and 5 grams, respectively. They had major symptoms of pain in the abdomen, and vomiting, and two patients had yellowish discoloration of the eyes. Patients were admitted to the Intensive care unit, gastric lavage was given with potassium permanganate and supportive medical management with N-acetyl cysteine (NAC) 150 mg/kg over 30 minutes followed by 50 mg/kg over 4 hours followed by 100 mg/kg over 16 hours diluted in 5% dextrose, and Vit-K 30 mg was given. Despite manageable treatment, two patients (Cases 1 and 2) developed toxic hepatitis (TH) and acute liver failure (ALF). Along with NAC and Vit-K, both patients (Cases 1 and 2) received multiple fresh frozen plasma (FFPs) transfusions. Daily clinical evaluation like vitals, detailed general examination, and laboratory monitoring with liver function test, PT, and INR was done. Serial charts of biological markers were plotted for timely intervention. All patients received psychiatric evaluation and counseling and were discharged in stable condition.

## Discussion

Rattol paste contains 3% of yellow phosphorus, a more poisonous substance than red phosphorus. It can get ingested through the skin, mucus membrane, respiratory and gastrointestinal epithelium [7]. Self-destructive or incidental ingestion leads to intoxication [8]. After assimilation, it is circulated to all tissues, especially the liver, and the peak levels are achieved at two to three hours of harmful oral ingestion. Bile salts are important for the absorption of phosphorus. Because of water content and low oxygen tension, phosphorus stays stable in the stomach for a longer period. It is categorized as a highly lethal rodenticide. Phosphorus is a general protoplasmic poison causing heart, hepatic, renal, and multiorgan dysfunction. There is no specific antidote for yellow phosphorus. Treatment is focused on elimination of the poisonous substance and adjuvant treatment. Gastric lavage with potassium permanganate is favored to convert phosphorus into harmless oxides [2]. Careful monitoring of hepatic and renal functions and management of their failure is required. In our study, two cases presented with acute liver failure showed improvement with the given treatment and meticulous monitoring with favorable outcomes. Various systems can be affected like the hepatic, renal, cardiac, and central nervous system, so daily clinical evaluation should be done [4,5].In our opinion, creating awareness, among the general population, and promoting mental health to prevent suicidal attempts is urgently required. It is the most ideal choice to diminish mortality from such poisonings. Lethal dosage and delayed presentation to a hospital can prompt liver dysfunction, liver failure, multiple organ failure, and serious illness [9]. Rodenticides should not be available easily. Timely intervention, including liver transplantation for suitable candidates, could lead to favorable outcomes despite complications. Strict measures against the easy availability of poison need to be regulated.

## Conclusions

Disseminating awareness about this poison among people and early healthcare, especially in rural regions, is needed. Early identification and prompt management lead to a better prognosis and can prevent complications of hepatic failure. Administrative and legal measures against simple accessibility of this harmful substance should be directed. Identifying suitable patients for liver transplantation can reduce mortality.
